# Piriformis Syndrome in Fibromyalgia: Clinical Diagnosis and Successful Treatment

**DOI:** 10.1155/2014/893836

**Published:** 2014-09-22

**Authors:** Md Abu Bakar Siddiq, Moshiur Rahman Khasru, Johannes J. Rasker

**Affiliations:** ^1^Physical Medicine and Rehabilitation Department, Feni Diabetes Hospital (FDH), Feni 3900, Bangladesh; ^2^Physical Medicine and Rehabilitation Department, BSMMU, Dhaka 1000, Bangladesh; ^3^Rheumatology Department, Faculty of Behavioral Sciences, University of Twente, P.O. Box 217, 7500 AE Enschede, The Netherlands

## Abstract

Piriformis syndrome is an underdiagnosed extraspinal association of sciatica. Patients usually complain of deep seated gluteal pain. In severe cases the clinical features of piriformis syndrome are primarily due to spasm of the piriformis muscle and irritation of the underlying sciatic nerve but this mysterious clinical scenario is also described in lumbar spinal canal stenosis, leg length discrepancy, piriformis myofascial pain syndrome, following vaginal delivery, and anomalous piriformis muscle or sciatic nerve. In this paper, we describe piriformis and fibromyalgia syndrome in a 30-year-old young lady, an often missed diagnosis. We also focus on management of the piriformis syndrome.

## 1. Introduction

The piriformis, a “pear shaped” (Latin piriformis means pear shape) skeletal muscle underneath the gluteal muscles originates in the pelvic cavity (anterior to sacrum, sacroiliac joint capsule, superior margin of greater sciatic notch, and sacrotuberous ligament), runs through the greater sciatic notch, and inserts outside the pelvic cavity (top of greater trochanter of the femur). During its passage it divides the greater sciatic notch into two compartments: superior and inferior. The muscle externally rotates the corresponding femur in hip extension and abducts the femur in hip flexion [1]. The piriformis syndrome (PS) is a clinical entity related to piriformis muscle where patients usually present with localized buttock and radiating pain in thigh and or leg. The typical physical examination findings include tenderness on the buttocks from the sacrum to greater trochanter, piriformis tenderness on pelvic/rectal examination, or pain provocation by FAIR (flexion, adduction, and internal rotation) test, Pace sign, Freiberg test, and so forth [2]. Fibromyalgia (FMS) is an idiopathic, chronic, nonarticular pain syndrome defined by widespread musculoskeletal pain but is believed to involve genetic, psychological, and environmental factors [3]. Patients usually complain of widespread body ache with associated fatigue, anxiety, sleep disturbance, morning stiffness, headache, tingling/numbness, cognitive disturbance, and so forth [3]. Along with these clinical features there should be generalized tender points on palpation to satisfy American College of Rheumatology (ACR) 1990 criteria for fibromyalgia [4]. FMS and PS are predominant in women [2, 4]. We could not find any literature describing both these conditions in the same patient. In this paper we describe the occurrence of both piriformis and fibromyalgia syndrome in a patient, with the intention to increase awareness among physicians of this combination. Stress is also given on management of the piriformis syndrome.

## 2. Case Report

A 30-year-old Asian women, housewife, presented with complaints of multiple body area pain for the last few months, in left and right side of the body and upper and lower parts, hardly improving with traditional NSAIDs (like naproxen, etoricoxib, diclofenac) and analgesics (paracetamol, tramadol). She also complained of pain in multiple large and small joints with morning stiffness that lasted for more than half an hour. There was associated fatigability. She had no joint swelling or limitation of joint movements. There was no history of significant hair loss, oral ulceration, altered bowel/bladder habits, headache, and so forth. Her menstrual and obstetric history was uneventful. General and systemic examination was unremarkable except that 14 out of 18 ACR fibromyalgia tender points were extremely painful. Laboratory tests were normal including complete blood count; Hb-12 g/dL, ESR −20 mm 1st hr, TC-4500/cmm; C-reactive protein negative; anti-CCP and ANA negative. Serum lipids, thyroid, and viral profile for hepatitis B and C also were normal. After interpreting both clinical and laboratory information she was classified as fibromyalgia. The patient was treated with amitriptyline (10 mg) (at night) and fluoxetine (20 mg) (at morning). Aerobic exercise in the form of swimming was also encouraged. After 3 weeks of follow-up, she reported significant improvement of fatigue and body ache except a deep seated right gluteal pain and discomfort. The pain was also radiating in her right thigh and leg with tingling in the same distribution. The pain was aggravated on sitting, lying on her right side, forward bending, and walking. Sometimes sitting was so disturbing that she could not sit more than 30 minutes on a chair. She could not memorize any recent/previous trauma over the gluteal region, significant history of fall, or traffic accidents that did impact on her lower back. She had no history of vaginal delivery in her recent past.

**Figure 1:**
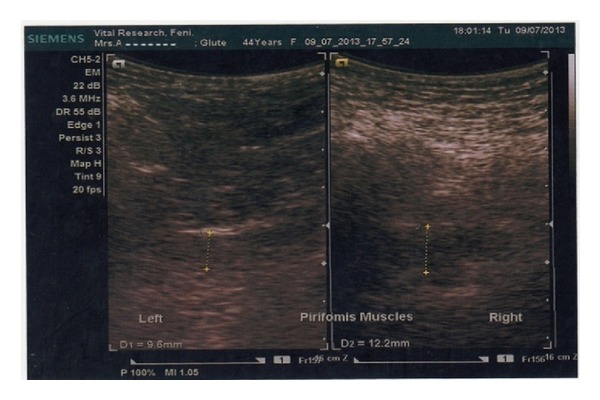
High frequency diagnostic ultrasonogram of both gluteal regions illustrates piriformis muscle thickness (right 12.2 and left 9.4 mm).

**Figure 2:**
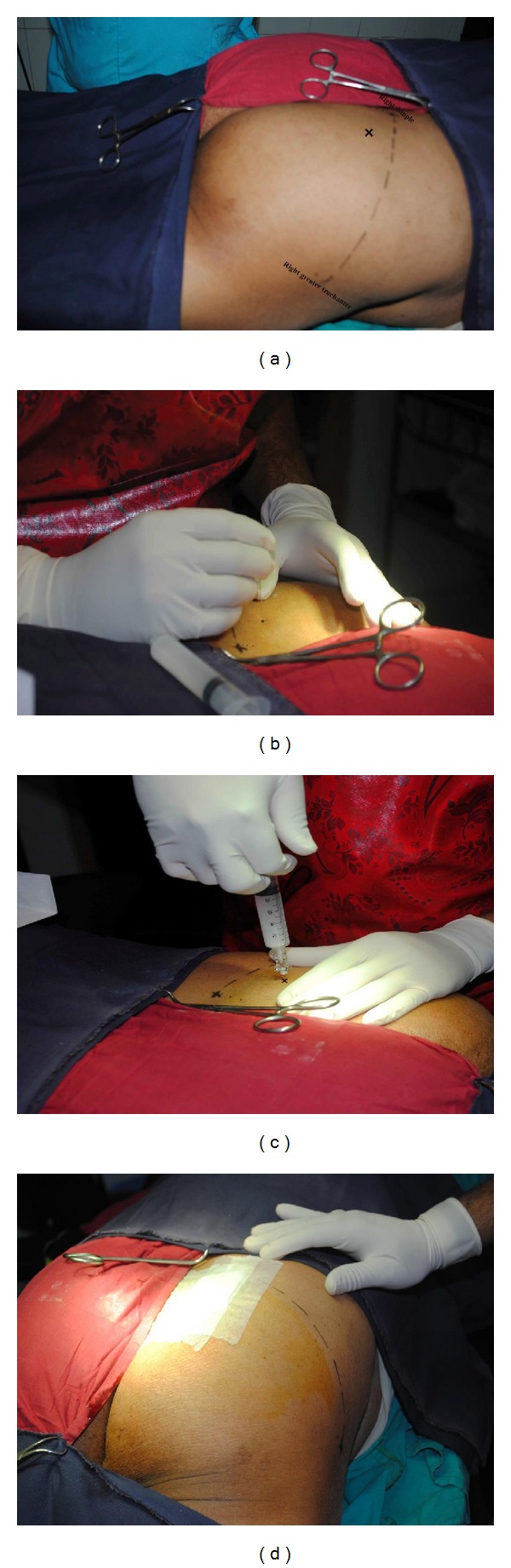
Intralesional steroid injection in right piriformis muscle. (a)** x** indicates point of needle entry at 1.5 cm lateral and 1.2 cm caudal to the lower 3rd of right sacroiliac joint and dotted line from right dimple of Venus to right greater trochanter runs parallel with superior margin of the right piriformis muscle; (b) after infiltration with 1% lidocaine spinal needle is placed; (c) 10 cc disposable syringe with injection methylprednisolone (40 mg/mL), 3 mL 1% lidocaine, and 2 mL 0.25% bupivacaine is in situ; (d) local gauze bandage after the procedure.

On physical examination, tenderness was elicited over the right gluteal region mostly at the greater sciatic notch. The pain was provoked by FAIR test, Pace sign, and digital rectal examination. Nervous system examination of the lower limbs revealed no abnormalities. MRI lumbosacral spine revealed only discs bulging at L4-5-S1 levels. There was no disc degeneration or nerve roots compression. Ultrasonography (USG) (Siemens Acuson X300 premium edition, transducer: CH 5-2, Germany) of the gluteal region revealed asymmetry of the piriformis muscle thickness (right 12.2 and left 9.4 mm) (Figure 1). At that time her pain was quantified as 9/10 on a visual analogue scale (VAS, 0–10 cm) for pain. Along with oral medications she was taught how to do piriformis muscle stretching exercise. After 3 weeks of follow-up, the patient still had the same complaints but with less intensity and her pain was 4/10 on the VAS. Finally, we decided to put intralesional (IL) methylprednisolone 40 mg at the right piriformis muscle (Figure 2) and counseling was done accordingly. After that treatment, her buttock pain was found to be improved and it was felt only after sitting for longer than two hours.

### 2.1. Technique of Piriformis Muscle Injection (Figures 2(a)–2(d))

After obtaining informed consent the patient with PS is placed in the prone position. The buttock area on the right side is sterilized with povidone-iodine USP (10%) and draped in a sterile fashion. Using surface anatomy the lower one-third of the right sacroiliac joint is identified [5]: at the level of dimple is the middle of the sacroiliac joint and just inferior to the dimple, close to the greater sciatic notch is the lower part of sacroiliac joint. The skin over the gluteal region 1.5 cm lateral and 1.2 cm inferior to the lower sacroiliac joint is marked as the site of needle insertion. After skin infiltration with 1 mL of 1% lidocaine, a 22-gauge, 10 cm insulated needle is inserted perpendicular to the right piriformis muscle until it touches the ilium and is then withdrawn 1-2 mm to relocate it in the piriformis muscle. At this point, the patient is asked whether she experiences any buttock pain and whether it coincides with her usual pain. A 10 mL disposable syringe is prepared with methylprednisolone (40 mg/mL), 1% lidocaine (4 mL), and 0.25% bupivacaine (3 mL) and injected in the desired place (Figures 2(a)–2(d)). After the procedure, the patient is brought to the recovery room for one hour or until any leg numbness has subsided, or for a longer period when necessary.

## 3. Discussion

The piriformis syndrome is an elusive medical condition [6], one of the most common extraspinal causes of sciatica. The reported incidence rates for PS among patients with low back pain vary widely from 5% to 36%. It is more common in females than in males [2, 7]. Symptoms and clinical signs relate either directly or indirectly to muscle spasm and resulting sciatic nerve compression. Pain originating from the trochanteric bursa, sacroiliac joint, or facet joint may also be confused with this clinical scenario. Patients usually present with low back pain mostly over the buttock that aggravates on sitting more than 20–30 minutes. Some patients may present with sudden severe low back pain causing difficulty in bodily movements, others give a history of deep seated gluteal pain for longer periods of time that interfere with daily activities. There may be an associated tingling sensation in the lower limb or subjective heaviness of the same extremity. Patients also complain of difficulty when walking, rising from sitting position, cross-legged sitting, or ambulation [2, 8]. Though there is no single test specific for PS, the following tests are generally used to diagnose PS [2]: the Freiberg test, the FAIR test, the Pace sign, the Beatty test, and straight leg raising (SLR). Tenderness with palpation over the piriformis muscle is common. Patients may also experience tenderness in the sacroiliac joints and greater sciatic notch. Some patients have a palpable sausage shaped mass in the buttock caused by contracted piriformis muscle [2]. Findings with straight leg raising are variable in PS [2, 9].

The following medical conditions are frequently associated with complaints of the piriformis syndrome: (1) preceding fall, (2) direct gluteal trauma, (3) overuse of piriformis muscle, (4) LLD (leg length discrepancy), (5) lumbar spinal stenosis, (6) myofascial pain syndrome (MPS), (7) piriformis muscle infection, and (8) local invasion of piriformis muscle by cervical cancer [2, 6, 10–16]. After a fall or direct gluteal blow there may be localized hematoma followed by scarring in between sciatic nerve and small hip extensors. Sometimes piriformis muscle spasm may irritate the underlying sciatic nerve [6]. LLD can be subdivided into two etiological groups: a structural LLD defined as those associated with a shortening of bony structures and a functional LLD defined as those that are a result of altered mechanics of the lower extremities or spine. The most controversial musculoskeletal disorder associated with leg length discrepancy is low back pain [14]. Gait pattern may be altered or remain unchanged in leg length inequality. Sustained stress on piriformis muscle with resultant impact on both stance and swing phases can produce altered gait pattern in LLD [6]. Overuse of piriformis muscle can occur following unaccustomed long distance walking, running, repeated squatting, kneeling, cycling, and so forth [2]. Piriformis pyomyositis is an infective condition involving the piriformis muscle, a clinical scenario that may report following vaginal delivery and usually is associated with fever and raised inflammatory biochemical markers [10]. Association of piriformis syndrome and lumbar stenosis can be explained by double crush hypothesis [16].

Sometimes PS is due to myofascial pain syndrome involving piriformis muscle with taut band and trigger points (TrPs). Primary MPS often regards the typical overuse syndrome that is named for the structures involved or the common conditions that produce it. PS is an example of primary MPS due to existing TrPs in the contracted piriformis muscle. Although the myofascial pain syndrome is a localized painful muscle condition, sometimes it may present as widespread body pain due to spread of TrPs: (i) through axial kinetic chain; (ii) through the activation of TrPs in the overloaded or mechanically stressed muscle compensating for the dysfunction of other muscles in the functional muscle units. Sometimes the clinical picture of widespread MPS may be confused with FMS [13]. MPS and FMS may also coexist in the same patient and may share common pathophysiology [13, 15]. Central sensitization is important in the genesis of both FMS and MPS. It could explain both the physical findings and biomechanical changes that have been documented in fibromyalgia [13]. According to Gerwin 75% of FMS may have significant MPS at one or more times during the course of their illness. In MPS an increase in TrPs Ach release could result in sustained depolarization of the postjunctional membrane of the muscle fiber and produce sustained sarcomere shortening and contracture with increased local energy consumption and reduction of local circulation, producing local ischemia and hypoxia. The localized muscle ischemia stimulates the release of prostaglandins, substance P, bradykinin, capsaicin, serotonin, and histamine that sensitize afferent nerve fibers in muscle. Under pathologic conditions, convergent connections from deep afferent nociceptors to dorsal horn neurons are facilitated and amplified in the spinal cord with pain referral beyond the initial nociceptive region owing to spreading of central sensitization to adjacent spinal segments. At the level of the central nervous system, spinal neuroplastic changes in the second order neuron pool produce a long lasting increase in the excitability of nociceptor pathways. Neurotransmitters involved in the process of central sensitization include substance P, N-methyl-D-aspartate, glutamate, and nitric oxide. In addition, there may be impairments in supraspinal inhibitory descending pain control pathways. Like MPS, there is no significant peripheral pathology in FMS. Central sensitization is the most important CNS aberration in FMS with altered neurotransmitters in serum (decrease serotonin) and CSF (increase substance P) [2, 13].

Since our patient had none of the cited risk factors for PS, another possibility might be sought. Although both conditions may be present in a single individual, unrecognized and poorly managed MPS of piriformis muscle appears to be the causative agent of FMS in our case. Longstanding nociceptive stimuli in piriformis muscle could cause sensitization in the central nervous system which is a manifestation of neuroplasticity or the remodeling of central processes produced generalized body ache in our patient. Along with widespread body ache and generalized tender areas, the patient also had fibromyalgia symptoms; morning stiffness and fatigability. Pain was provoked in favor of piriformis syndrome using FAIR test, Pace sign, and localized tenderness over the right gluteal region. On digital rectal examination piriformis tenderness was also elicited during finger gliding over the right lateral pelvic wall. Right piriformis muscle was found to be thicker than the left one using high frequency ultrasonography of gluteal region that indicated some sorts of muscle spasm.

NSAIDs, analgesics, and muscle relaxants are being used for the management of PS. Nonpharmacological approaches may also be efficacious in managing this painful condition like therapeutic deep heating, piriformis muscle stretching, and therapeutic manipulation. According to Fishman et al. 79% patients with PS had symptom reduction with conservative approaches. When all these approaches have failed, intralesional (IL) injection lidocaine and corticosteroid or botulinum toxin (A/B) under high resolution USG or fluoroscope can be an alternative to manage this condition [2]. Where high resolution ultrasonogram/fluoroscope are not available, motor stimulation guided piriformis muscle injection using surface anatomy can be another option [2]. In a cadaveric study by Gonzalez et al. it was described that needle positioning at a distance of approximately 1.5 cm lateral and 1.2 cm caudal to the lower border of the sacroiliac joint can be used successfully for piriformis muscle injection [17]. To accelerate pain relief, along with oral medications our patient was successfully treated with intralesional steroid using surface anatomy followed by gradual graded stretching exercises for the piriformis muscle.

In conclusion, PS may be associated with fibromyalgia and this case report should make physicians aware of neurological aspects of fibromyalgia in which the piriformis muscle is involved. Coexistence of FMS and MPS is not uncommon, but to our best knowledge this is the first clinical report describing MPS as piriformis syndrome in a patient with fibromyalgia. We strongly believe that a case study is not enough for establishing etiological association between PS and FMS. So, we recommend further prospective multicenter studies to measure prevalence, association of piriformis syndrome in fibromyalgia.
